# *In Silico* Study of Coumarins: Wedelolactone as a Potential Inhibitor of the Spike Protein of the SARS-CoV-2 Variants

**DOI:** 10.1155/2023/4771745

**Published:** 2023-02-06

**Authors:** Saurav Katuwal, Siddha Raj Upadhyaya, Rishab Marahatha, Asmita Shrestha, Bishnu P. Regmi, Karan Khadayat, Saroj Basnet, Ram Chandra Basnyat, Niranjan Parajuli

**Affiliations:** ^1^Central Department of Chemistry, Tribhuvan University, Kirtipur, Kathmandu, Nepal; ^2^Department of Chemistry, Florida Agricultural and Mechanical University, Tallahassee, FL 32307, USA; ^3^Center for Drug Design and Molecular Simulation Division, Cancer Care and Research Center, Kathmandu, Nepal

## Abstract

Despite the rigorous global efforts to control SARS-CoV-2 transmission, it continues to pose a serious threat to humans with the frequent emergence of new variants. Thus, robust therapeutics to combat the virus are a desperate need. The SARS-CoV-2 spike (S) protein is an important target protein as it mediates the entry of the virus inside the host cells, which is initiated by the binding of the receptor-binding domain (RBD) to its cognate receptor, angiotensin-converting enzyme 2 (ACE-2). Herein, the inhibition potential of several naturally occurring coumarins was investigated against the spike proteins of SARS-CoV-2 variants using computational approaches. Molecular docking studies revealed 26 coumarins with better binding energies than the reference ligands, molnupiravir and ceftazidime, against the S-RBD of the omicron variant. The top 10 best-docked coumarins were further analyzed to understand their binding interactions against the spike proteins of other variants (wild-type, Alpha, Beta, Gamma, and Delta), and these studies also demonstrated decent binding energies. Physicochemical, QSAR, and pharmacokinetics analyses of the coumarins revealed wedelolactone as the best inhibitor of the spike protein with ideal Lipinski's drug-likeness and optimal ADMET properties. Furthermore, coarse-grained molecular dynamics (MD) simulation studies of spike protein-wedelolactone complexes validated the stable binding of wedelolactone in the respective binding pockets. As an outcome, wedelolactone could be utilized to develop a potent drug candidate against COVID-19 by blocking the viral entry into the host cell.

## 1. Introduction

All viruses change over time, and the severe acute respiratory syndrome coronavirus 2 (SARS-CoV-2), the causative agent of the ongoing devastating pandemic, coronavirus disease 2019 (COVID-19), is no exception. Since its first outbreak near the end of 2019 in Wuhan, China, it has been persistently emerging in the form of distinct variants, evolving via mutations, claiming the lives of more than 6 million people, and infecting more than 500 million people to date [1]. Although several vaccines and therapeutics are currently available, their long-term efficacies on new variants are yet to be evaluated, and the fact that certain variants of SARS-CoV-2 can circumvent the neutralizing antibodies produced by the vaccines further worsens the scenario [2]. Thus, robust therapeutics to combat the virus are a desperate need.

The World Health Organization (WHO) has classified the SARS-CoV-2 variants into three categories: variants of concern (VOCs), variants of interest (VOIs), and variants under monitoring (VUMs) [3]. Previously reported SARS-CoV-2 VOCs, Alpha (B.1.1.7), Beta (B.1.351), Gamma (P.1), and Delta (B.1.617.2), illustrated that mutations, for example, D614G, that increase the virus's transmissibility, have a major evolutionary benefit [4, 5]. On November 26, 2021, a new variant designated as Omicron (B.1.1.529) was added to the list of VOCs owing to its increased transmissibility, a large number of mutations, and increased risk of reinfection, indicating a detrimental change in COVID-19 epidemiology [6]. There is much concern regarding the speed of transmission of the Omicron variant around the globe, even among fully immunized individuals. Furthermore, the spike protein of the Omicron variant carries 3–5 times more mutations than any of the previous SARS-CoV-2 variants [7]. The mutations occurring in the S-RBD of SARS-CoV-2 that are variants of concern in comparison to the wild-type variant are shown in Table S1 of the supplementary file. Mutations can occur in any part of the viral genome. However, mutations occurring in the spike protein, specifically within its receptor-binding domain, i.e., from amino acid residue Arg319 to Phe541, are critical for infectivity [8] as S-RBD is directly involved in host recognition and interaction with the host receptor, human angiotensin-converting enzyme 2 (ACE-2) [9]. By binding to human ACE-2, the SARS-CoV-2 spike protein plays a crucial role in viral entry into the host cell, and this unique interaction provides a viable therapeutic target for drug discovery. Progress in drug discovery rests to a great extent on identifying possible targets for medication, and in the present work, spike proteins of different variants of SARS-CoV-2 were chosen as a target to develop therapeutics against COVID-19.

Nature has magnanimously supplied us with a plethora of natural products with a Catholic range of structural and chemical properties adored by low toxicity and minimum side effects. Natural products and their derivatives such as polyphenols, terpenoids, coumarins, flavonoids, quinones, and alkaloids have been utilized for treating and preventing a variety of disorders including viral infections [10]. Among these, coumarins have gained particular attention as effective antiviral agents against several viruses such as the human immunodeficiency virus (HIV) [11], influenza A virus [12], hepatitis B and C viruses [12, 13], herpes simplex virus (HSV) [14], poliovirus [15], chikungunya virus, and dengue virus [16]. Several investigations have found that naturally occurring coumarins possess antiviral properties by blocking the function of several proteins of the virus, such as integrases, proteases, reverse transcriptase, and DNA polymerase, as well as impeding viral entrance [17, 18]. The reported antiviral activities of selected natural coumarins are given in Table S2 of the supplementary file.

In the present research, *in silico* molecular docking of 42 natural antiviral coumarins followed by their physicochemical, QSAR, and pharmacokinetic analyses, molecular target prediction, and coarse-grained MD simulation studies were performed to identify the potent coumarins against the spike proteins of SARS-CoV-2 variants, focusing on the latest VOC, the Omicron variant.

## 2. Materials and Methods

### 2.1. Ligand Selection and Preparation

Forty-two natural coumarins, with reported inhibition activities against different viruses along with molnupiravir and ceftazidime as reference ligands, were selected for the study. The 3D conformer of all the ligands was retrieved from PubChem (https://pubchem.ncbi.nlm.nih.gov/) in SDF (structure data format) and converted to PDB (protein data bank) format using Open Babel software. In addition, all the coumarin structures were drawn in ChemDraw Pro version 16.0 and verified through the ChemSpider database (https://www.chemspider.com/). The ligands in PDB format were then imported into AutoDock 1.5.6 workspace, which by default added PDBQT charges (protein data bank, partial charge, Q, atom type, and T). Finally, the prepared ligands were saved in the PDBQT format.

### 2.2. Protein Selection and Preparation

The X-ray crystal structures and the Cryo-EM structure (Omicron variant) of the spike proteins of SARS-CoV-2 variants (wild-type, Alpha, Beta, Gamma, Delta, and Omicron) with respective PDB IDs (6M0J, 7EKF, 7EKG, 7EKC, 7WBQ, and 7T9J) and respective resolutions (2.45 Å, 2.85 Å, 2.63 Å, 2.80 Å, 3.34 Å, and 2.79 Å) were retrieved from the RCSB Protein Data Bank (https://www.rcsb.org/) in the PDB format. The proteins were then imported into AutoDock 1.5.6 workspace, and the unnecessary chains, heteroatoms, and water molecules were deleted from the proteins. Furthermore, polar hydrogens and the Kollman charges were added. Finally, the prepared proteins were saved in the PDBQT format.

### 2.3. Binding Site Identification

Several previous studies on the SARS-CoV-2 spike proteins and the human ACE-2 receptor have already identified the crucial binding site residues in the S-RBDs of the wild-type, Alpha, Beta, Gamma, and Delta variants [19–22]. In addition to these, all 15 mutations reported on Omicron S-RBD [7] (Table S1, supplementary file) were also considered potential binding sites for screening against our ligands in the present work.

### 2.4. Molecular Docking

Molecular docking studies were carried out using the popular open-source software AutoDock Vina 1.5.6 [23]. The binding site residues of the S-RBDs of different variants were enclosed inside the grid boxes of varying dimensions. For 7T9J, the grid box was created with the size of 56 × 68 × 54 *xyz* points, and the grid spacing was adjusted to 0.675 Å, whereas for the rest of the spike proteins, the default dimensions, i.e., the size of 40 × 40 × 40 *xyz* points and the grid spacing of 0.375 Å, were used. For 7T9J, the grid centers were set at *x*, *y*, and *z* dimensions of 198.713, 179.110, and 273.829, respectively. For 6M0J, the grid centers were set at *x*, *y*, and *z* dimensions of −34.704, 23.660, and 2.916, respectively. For 7EKF, the grid centers were set at *x*, *y*, and *z* dimensions of −35.605, 18.218, and 11.957, respectively. For 7EKG, the grid centers were set at *x*, *y*, and *z* dimensions of −35.901, 17.592, and 7.586, respectively. For 7EKC, the grid centers were set at *x*, *y*, and *z* dimensions of −38.879, 21.924, and 8.237, respectively. Finally, for 7WBQ, the grid centers were set at *x*, *y*, and *z* dimensions of 30.998, 38.399, and 67.645, respectively. Next, these grid box attributes as well as the protein and ligand information were saved in a configuration file with the default value for exhaustiveness, and docking was executed. The visualization of the ligand-protein binding interactions was performed via the BIOVIA Discovery Studio visualizer.

### 2.5. Validation of the Docking Protocol

To ensure the accuracy and reliability of docking as well as to get rid of false-positive results, the docking protocol was validated using the methods of redocking and superimposition [24, 25]. First, one of the reference ligands, molnupiravir, was docked within the designated binding sites of the Omicron spike protein. The docked molnupiravir-RBD complex with the lowest energy pose was imported to the Discovery Studio Visualizer workspace where the ligand-protein interactions, as well as the binding site attributes, were noted. Then, the docked molnupiravir was detached from the complex and redocked using the same binding site attributes. The lowest energy poses of the redocked ligand and the former ligand were then superimposed to compute the all-atom RMSD (root-mean-square deviation) value. RMSD value ≤2 Å generally validates the docking protocol [26, 27].

### 2.6. Physicochemical, QSAR, and Pharmacokinetic Studies

The physicochemical, quantitative structure-activity relationship (QSAR), and pharmacokinetic properties of the natural coumarins that displayed good binding energies and interactions against the spike proteins of SARS-CoV-2 variants were analyzed *in silico*. The SMILES (Simplified Molecular Input Line Entry System) of the selected compounds were copied from PubChem and submitted to the respective servers. Lipinski's (Pfizer) rule of five was assessed via the SwissADME web server [28]. The drug score was assessed via OSIRIS Property Explorer software (https://www.organic-chemistry.org/prog/peo/), the pkCSM web server was used for ADMET (absorption, distribution, metabolism, excretion, and toxicity) analysis [29], the PASS online-Way2Drug server (https://way2drug.com/PassOnline) was used for the QSAR analysis, and the Swiss Target Prediction server [30] was used for molecular target prediction.

### 2.7. Molecular Dynamics Simulation Studies

MD simulations were performed using the CABS-flex 2.0 web server [31]. It utilizes the coarse-grained protein modeling tool and obtains near-native dynamics of proteins from 10 ns (nanosecond) MD simulations. PDB files were uploaded to the server with the default settings. The results were analyzed based on root-mean-square fluctuations (RMSF) for the protein-ligand complexes and the respective ligand-free proteins.

## 3. Results and Discussion

### 3.1. Validation of the Docking Protocol

**Figure 1:**
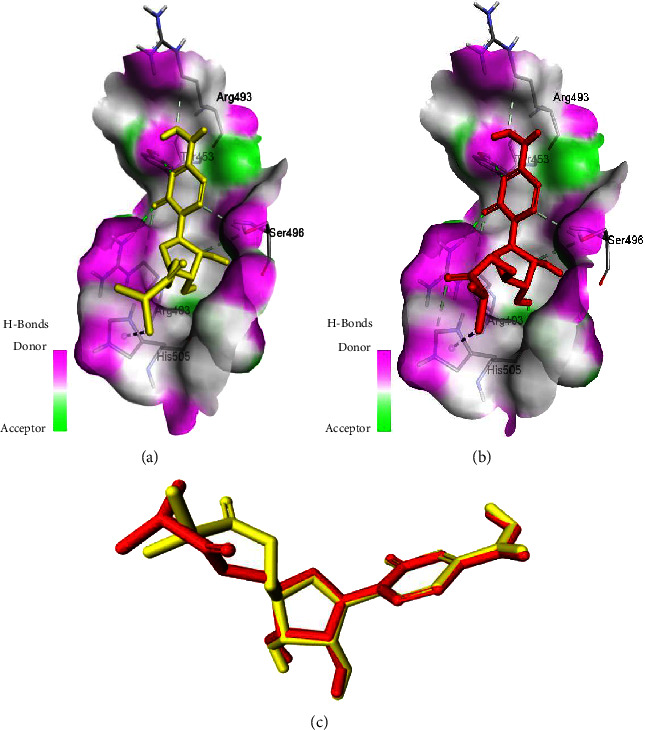
Validation of the docking protocol. Comparative analysis of the binding interactions for the first docked molnupiravir (yellow) (a) versus the redocked molnupiravir (red) (b) in the binding sites of Omicron S-RBD. Superimposition of the two ligands, yellow and red (c) (RMSD = 0.9845 Å).

After redocking the reference ligand molnupiravir into the binding sites of Omicron spike protein, the lowest energy pose of the redocked ligand displayed almost the same pattern of docking with the same binding energy value of −6.1 kcal/mol as compared to the former. Similarly, the ligand was found to be redocked exactly in the same binding pocket, interacting with the same amino acid residues of the Omicron spike protein (TYR 453, ARG 403, SER 496, ARG 493, and HIS 505) as shown in Figure 1. Superimposition of the lowest energy poses of the redocked ligand and the former ligand yielded a low RMSD value of 0.9845 Å, ensuring the validity and reproducibility of the docking protocol used and indicating that the protocol could be followed for subsequent docking studies.

### 3.2. Molecular Docking

**Figure 2:**
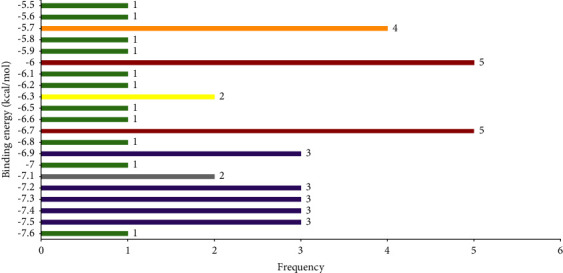
Frequency distribution of 42 natural coumarins and reference ligands over the range of binding energies when docked against the Omicron S-RBD.

**Figure 3:**
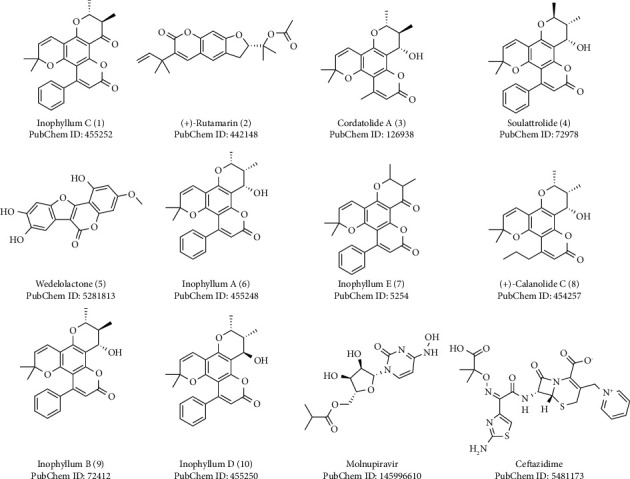
Chemical structures of the top 10 best-docked coumarins and the reference ligands.

All the selected 42 natural coumarins along with the reference ligands, molnupiravir and ceftazidime, were investigated through molecular docking studies in the binding sites of the Omicron spike protein. Out of 42 natural coumarins, 26 showed lower binding energies ranging from −6.6 to −7.6 kcal/mol than both the reference ligands, molnupiravir (−6.1 kcal/mol) and ceftazidime (−6.5 kcal/mol) (Table S3, supplementary file). The frequency distribution of 42 natural coumarins and reference ligands over the range of binding energies when docked against the Omicron S-RBD is depicted in Figure 2. The lower the binding energy, the higher the binding affinity and stability of the complex; as a result, the stronger is the inhibition [32, 33]. Out of the 26 natural coumarins, the top 10 best-docked coumarins with binding energies ranging from −7.3 to −7.6 kcal/mol (Table 1 and Figure 3) were selected for further study.

**Figure 4:**
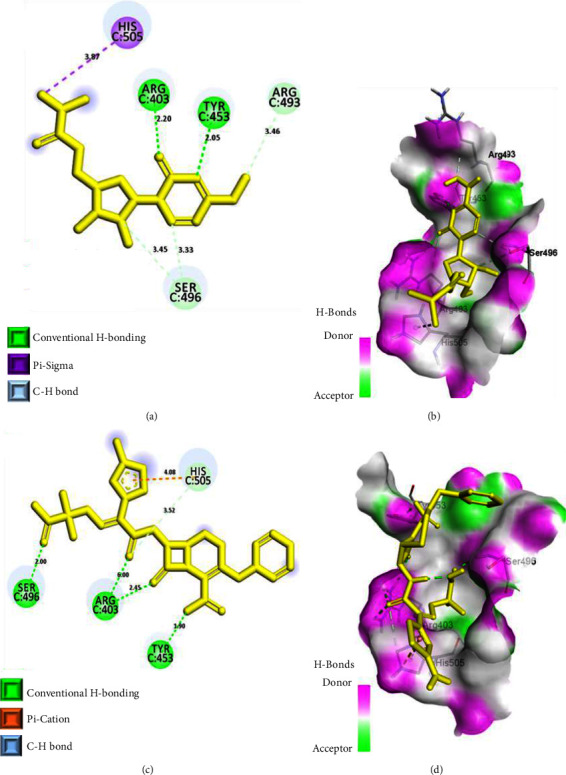
Binding interactions of reference ligands: molnupiravir (a, b) and ceftazidime (c, d) in the binding sites of Omicron S-RBD. (a, c) 2D-binding interaction diagrams. (b, d) 3D-binding interaction diagrams. Ligands are displayed as a yellow stick model. Protein residues in 3D diagrams are displayed as an atom-type color stick model.

As evident from Table 1 and Figures 4(a) and 4(b), molnupiravir, an oral antiviral drug, authorized by the FDA for emergency use against SARS-CoV-2 [34] displayed remarkable binding interactions with the Omicron spike protein, albeit with a relatively lower binding affinity. It showed interactions with three mutated residues in the binding region: two carbon-hydrogen bonding interactions with SER 496, a carbon-hydrogen bonding interaction with ARG 493, and a pi-sigma interaction with HIS 505. In addition, it was stabilized in the binding pocket through two hydrogen bonding interactions: one with TYR 453 (2.05 Å) and the other with ARG 403 (2.20 Å).

Likewise, another reference ligand, ceftazidime, a spike protein inhibitor [35], demonstrated similar binding interactions as molnupiravir with the Omicron spike protein but with a relatively higher binding affinity (−6.5 kcal/mol). It mediated interactions with two mutated residues in the binding region: a hydrogen bonding interaction with SER 496 (2.00 Å) and a pi-cation and a carbon-hydrogen bonding interaction with HIS 505. Furthermore, it was stabilized in the binding pocket through two hydrogen bonding interactions with ARG 403 (2.45 Å and 6.00 Å) and a hydrogen bonding interaction with TYR 453 (1.90 Å) (Figures 4(c) and 4(d)).

**Figure 5:**
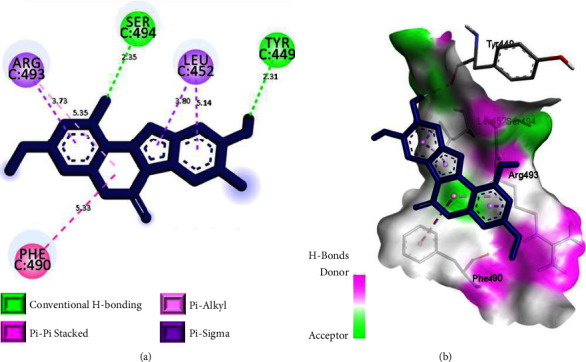
Binding interactions of wedelolactone in the binding sites of Omicron S-RBD. (a) 2D-binding interaction diagram. (b) 3D-binding interaction diagram. Ligands are displayed as a dark blue stick model. Protein residues in 3D diagrams are displayed as an atom-type color stick model.

Among the top 10 best-docked coumarins (Table 1), wedelolactone, a naturally occurring coumarin in *Wedelia calendulacea* [36], displayed the greatest number of binding interactions with the critical binding residues of Omicron spike protein, equivalent to that of the reference ligands. It also possessed low binding energy (−7.4 kcal/mol) indicating higher binding affinity towards the receptor. Similar to molnupiravir, wedelolactone interacted with the mutated residue ARG 493 in the binding region. However, unlike molnupiravir, the pi-electron cloud of the benzene ring in wedelolactone mediated a pi-sigma interaction with ARG 493, which in turn interacted with the pi-electron cloud of *⍺*-pyrone ring through a pi-alkyl interaction, indicating different modes of inhibition. In addition, wedelolactone demonstrated two hydrogen bonding interactions with crucial binding sites of Omicron S-RBD, one with TYR 449 (2.31 Å) and the other with SER 494 (2.35 Å). Interestingly, SER 494 in the S-RBD has also been identified as a crucial binding residue in stabilizing the reference ligand ceftazidime [35]. The pi-electron cloud of *⍺*-pyrone ring in wedelolactone further interacted with PHE 490 through a pi-pi stacking interaction. Furthermore, the side chain ring of benzofuran showed two pi-sigma interactions with LEU 452 (Figure 5).

The binding interaction analysis indicated that wedelolactone was well-docked inside the binding pocket of the Omicron spike protein through hydrogen bonding and hydrophobic interactions (pi-pi stacking, pi-sigma, and pi-alkyl). This led us to deduce that H-bonding and hydrophobic interactions are imperative in stabilizing the docked complex. Hydrogen bonding is crucial for protein-ligand binding stability, with the optimum bond distance between H-donor and H-acceptor atoms being less than 3.5 Å [36]. Additionally, the optimized hydrophobic interactions highly favor the tight binding of ligands into the binding pockets of proteins [37]. In our study, the hydrogen bond distances involved for all the selected coumarins were found to be below 3.5 Å, indicating a strong H bond between the receptor and ligands.

**Figure 6:**
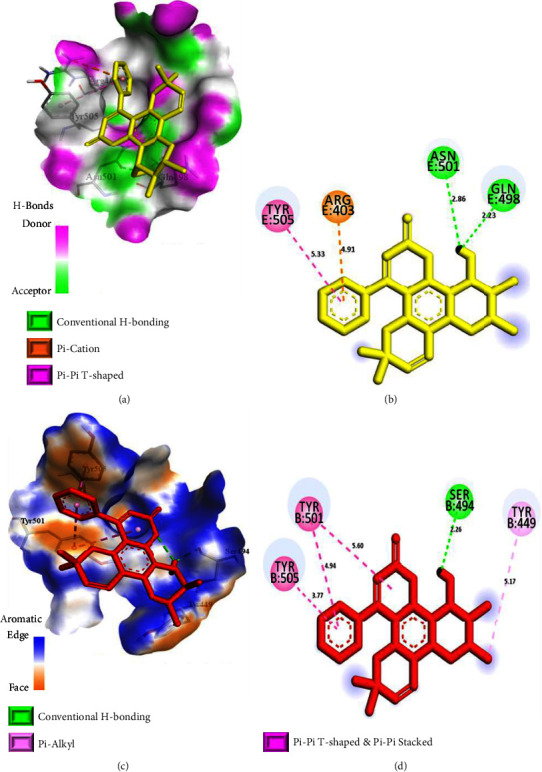
Binding interactions of inophyllum B (a, b) and inophyllum D (c, d) in the binding sites of the spike proteins of the wild-type variant and the Alpha variant, respectively. (a, c) 3D-binding interaction diagrams. (b, d) 2D-binding interaction diagrams. The protein surface of the wild-type variant is depicted as magenta for H-bond donors and green for H-bond acceptors, while the protein surface of the Alpha variant is depicted as orange for aromatic ring faces and blue for aromatic ring edges.

**Figure 7:**
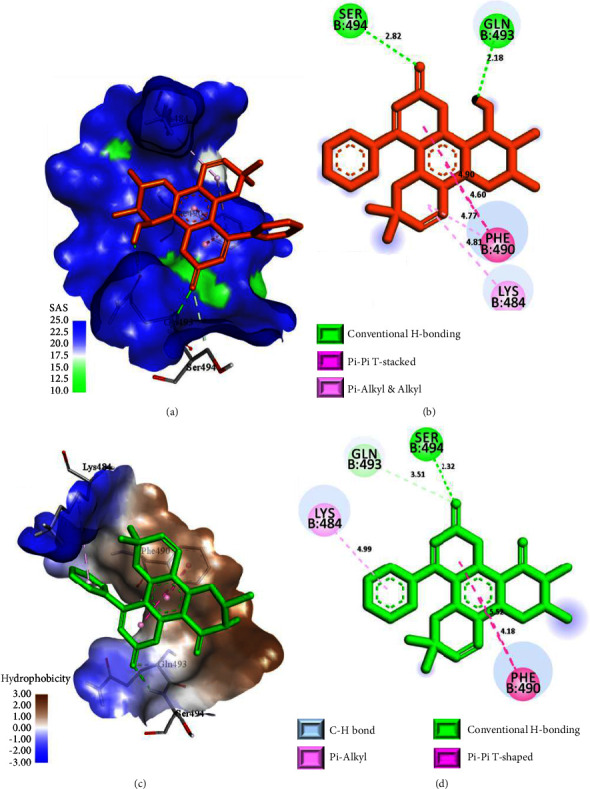
Binding interactions of soulattrolide (a, b) and inophyllum E (c, d) in the binding sites of the spike proteins of the Beta variant and the Gamma variant, respectively. (a, c) 3D-binding interaction diagrams. (b, d) 2D-binding interaction diagrams. The protein surface of the Beta variant is depicted as blue for exposed surfaces and green for buried surfaces, while the protein surface of the Gamma variant is depicted as blue for hydrophilic surfaces and brown for hydrophobic surfaces.

**Figure 8:**
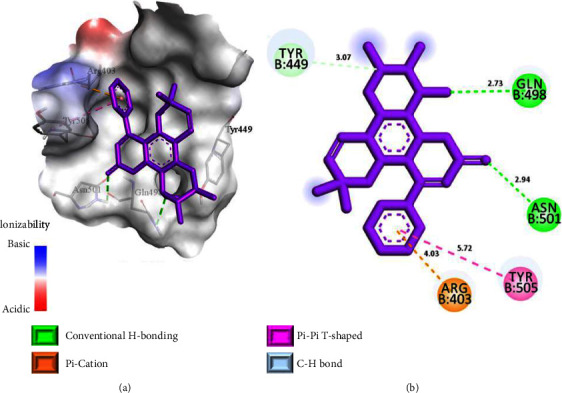
Binding interactions of soulattrolide (a, b) in the binding sites of the spike protein of the Delta variant. (a) 3D-binding interaction diagram. (b) 2D-binding interaction diagram. The protein surface of the Delta variant is depicted as blue for basic surfaces and red for acidic surfaces.

To further explore the inhibition efficacies of selected coumarins, the top 10 best-docked coumarins were further analyzed for their binding interactions against the spike proteins of other variants. Notably, also with other variants, the selected coumarins exhibited promising binding energies and interactions, strengthening their potential as effective spike protein inhibitors (Table 2 and Figures 6–8). Thus, binding interaction analysis of our top 10 best-docked coumarins with spike proteins of several SARS-CoV-2 variants revealed a consistent and precise mode of binding involving the key residues of spike proteins, which may impede the binding of the spike protein to the human ACE-2 receptor, interfering with further viral entrance, and eventually inhibiting the binding of the spike protein to its receptor [20]. Hence, the top 10 best-docked coumarins were selected for further physicochemical, QSAR, and pharmacokinetic analyses.

### 3.3. Physicochemical, QSAR, and Pharmacokinetic Studies

The main reasons for the high attrition rates of drug candidates in pharmaceutical industries and costly failures in drug development are due to their poor physicochemical and pharmacokinetic profiles [38]. Therefore, these key requirements must be thoroughly investigated at the preliminary stages of the drug development process. In this work, Lipinski's (Pfizer) Rule of Five, drug score, QSAR analysis, and ADMET analysis of the selected compounds were carried out.

#### 3.3.1. Lipinski's RO5 and Drug Score

Lipinski's (Pfizer) Rule of Five (RO5) is a pioneering physicochemical filter that relates the physicochemical parameters of drugs with their pharmacokinetic properties and examines the drug's oral bioavailability [39]. The drug score integrates lipophilicity, molecular weight, drug-likeness, solubility, and toxicity concerns into one convenient value that may be used to assess a compound's overall ability to be approved as a drug. All the selected compounds except for ceftazidime passed Lipinski's RO5 with no violation of Lipinski's drug-likeness parameters, and all of them were predicted to have a positive drug score (Table 3**)**. Among the tested coumarins, the highest drug score was predicted for wedelolactone (0.30), which was better than that of molnupiravir (0.18) but lower than that of ceftazidime (0.66). A negative drug score usually indicates that a compound is unlikely to be developed into a drug [20]. The highest drug score of wedelolactone may be partly attributed to the fact that it has the lowest molecular weight, which enhances its rate of absorption, transportation, and diffusion [20]. Additionally, the low lipophilicity (CLogP) value of wedelolactone (2.09) makes it highly water-soluble, thereby increasing its absorption and bioavailability even through other parenteral routes [28, 40]. The negative CLogP values of the reference ligands imply their preferred solubility in water. Since all the selected coumarins passed Lipinski's RO5 and drug score tests, they were further analyzed for their QSAR and pharmacokinetic properties.

#### 3.3.2. QSAR Analysis

**Figure 9:**
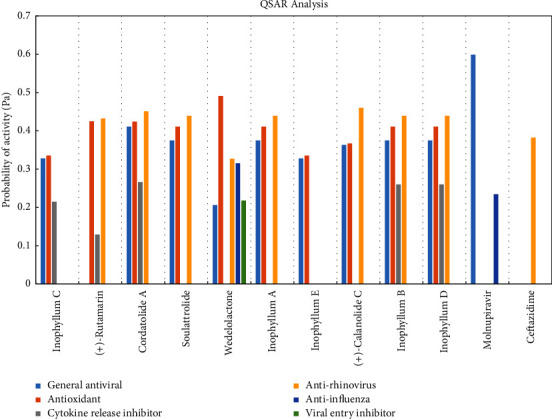
PASS predictions for the biological activities of the compounds based on QSAR.

All the selected coumarins along with the reference ligands were subjected to the PASS online server for QSAR analysis [41] to predict their antiviral, antioxidant, and cytokine release inhibitor activities. Owing to the striking similarities in the structural and functional homology of coronavirus with influenza and rhinovirus [42], the anti-influenza and antirhinovirus activities of the selected compounds were also investigated in the present work along with their general antiviral and viral entry inhibition properties. The PASS server predicts the biological activities of a compound based on its structural features in terms of probability (Pa and Pi: probability to be active and inactive, respectively). As expected, the reference ligand molnupiravir had the highest probability of being an active general antiviral agent (Pa = 0.599), whereas another reference ligand, ceftazidime, displayed only a moderate probability of being an active antirhinovirus agent (Pa = 0.382) (Figure 9). Although wedelolactone displayed the lowest probability of being an active general antiviral agent, it demonstrated decent probabilities of being an active anti-influenza, antirhinovirus, and viral entry inhibitor agent. Moreover, it also displayed the highest probability of being an active antioxidant (Pa = 0.491). Predictions were obtained only for 5 coumarins in terms of cytokine release inhibitory activity. Nevertheless, these coumarins displayed decent probabilities of being active cytokine release inhibitors, indicating their ability to impede cytokine storm in COVID-19 patients, preventing the failure of vital organs and unprecedented death [43].

#### 3.3.3. ADMET Analysis

The pkCSM web server was used to conduct an ADMET analysis of all the selected coumarins and the reference ligands (Table 4). All the selected coumarins displayed higher human intestinal absorption (HIA) (93.75%–100%) compared to both the reference ligands, molnupiravir (53.464%) and ceftazidime (16.74%), indicating a better absorption from the intestines after oral ingestion. Permeability-glycoprotein I (P-gp I) is an active drug efflux transporter that extrudes drugs and xenobiotics out of the cells, lowering absorption, bioavailability, and retention time, thereby protecting vital organs from toxic chemicals [44]. Six coumarins were predicted to be the substrate of P-gp I (Table 4), indicating that they will be actively pumped out of the biological membranes. However, since most of the substrates were also predicted to be the inhibitors of P-gp I, they are likely to inhibit the efflux pump and improve the delivery and bioavailability of drugs.

Regarding distribution analysis, blood-brain barrier (BBB) permeability was assessed. BBB permeability is an essential requirement for the central nervous system (CNS) active drugs, favoring their uptake from the bloodstream into the brain. However, for non-CNS-active drugs, their uptake into the brain may induce CNS toxicity. The lowest BBB permeability was predicted for wedelolactone (−1.35) followed by ceftazidime (−1.32) and molnupiravir (−1.06). For the rest of the compounds, BBB values ranged from −0.45 to 0.04, indicating a slight likelihood of crossing the BBB [29].

**Figure 10:**
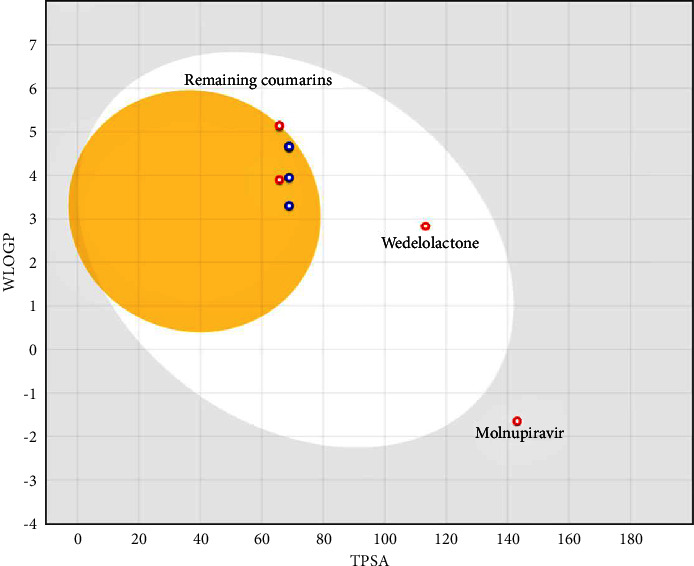
BOILED Egg model of the selected compounds. The compounds lying in the white region of the egg are predicted to have a high probability of absorption through the human intestine, the compounds lying in the yellow (yolk) region of the egg are predicted to have a high probability of BBB permeation, and the compounds lying in the grey region outside the egg are predicted to have low HIA and limited BBB permeation [46]. Here, ceftazidime is out of range (TPSA = 244.76 Å²).

The HIA and the BBB permeability of the selected compounds were also assessed by the BOILED Egg model (Brain Or IntestinaL EstimateD permeation) (Figure 10). Interestingly, the depiction of the BOILED-Egg model was consistent with the quantitative predictions made by the pkCSM server, suggesting wedelolactone possesses optimal HIA and is relatively safer for the CNS.

Concerning metabolism analysis, the CYP3A4 parameter was assessed. CYP3A4 is a major isoenzyme involved in the oxidative biotransformation and metabolism of more than 60% of drugs and xenobiotics in humans [45]. All the selected coumarins were predicted to be the substrate of the CYP3A4 isoenzyme, suggesting their proper metabolism. However, some of the coumarins (inophyllum C, (+)-rutamarin, inophyllum E, and (+)-calanolide C) were predicted to inhibit CYP3A4. Inhibition of CYP3A4 is associated with the bioaccumulation of coadministered drugs, leading to drug-drug interactions (DDI) and related toxicity [28]. Hence, despite the decent binding energies of inophyllum C (−7.6 kcal/mol), (+)-rutamarin (−7.5 kcal/mol), and (+)-calanolide C (−7.3 kcal/mol) against the spike protein of the Omicron variant and inophyllum E (−7.8 kcal/mol) against the spike protein of the Gamma variant, they are limited by their metabolic incompatibility.

All the selected coumarins showed a higher total clearance value (0.51–0.91) ml/min/kg than both the reference ligands, molnupiravir (0.20 ml/min/kg) and ceftazidime (0.23 ml/min/kg), indicating their better excretion through hepatic and renal routes. A low total clearance value for the reference ligands suggests that they will be retained by the body for a longer period.

Similarly, all the selected compounds, except for cordatolide A, showed a negative Ames toxicity test, meaning that they are nonmutagenic. A positive Ames toxicity test for cordatolide A suggests it is mutagenic and a potential carcinogen [46]. Thus, even though cordatolide A possessed the second-best binding energy against the Omicron spike protein (−7.5 kcal/mol), it was restrained by its carcinogenicity risks. Likewise, in the hepatotoxicity test, only 4 coumarins including (+)-rutamarin, cordatolide A, wedelolactone, and (+)-calanolide C were predicted to be nonhepatotoxic. Compounds with positive hepatotoxicity tests are often associated with drug-induced liver injury (DILI). Thus, despite the decent binding energies of inophyllum A (−7.4 kcal/mol) against the Omicron spike, inophyllum B (−7.4 kcal/mol) against the wild-type spike, inophyllum D (−7.5 kcal/mol) against the Alpha spike, and soulattrolide against Delta (−7.8 kcal/mol), Omicron (−7.5 kcal/mol), and Beta (−7.2 kcal/mol) spike proteins, they are circumscribed by their hepatotoxicity risks. Furthermore, all the selected coumarins showed higher LD_50_ values (2.41–3.18) mol/kg than both the reference ligands, molnupiravir (2.16 mol/kg) and ceftazidime (2.13 mol/kg), indicating their minimal lethal impacts.

Based on the results of *in silico* molecular docking followed by physicochemical, QSAR, and pharmacokinetic analyses, wedelolactone was found to be the best among the tested coumarins owing to its decent binding against the Omicron spike protein, highest drug score, highest antioxidant potential, decent antiviral activities, and optimal ADMET properties. Henceforth, only wedelolactone was considered for further studies.

#### 3.3.4. Molecular Target Prediction

**Figure 11:**
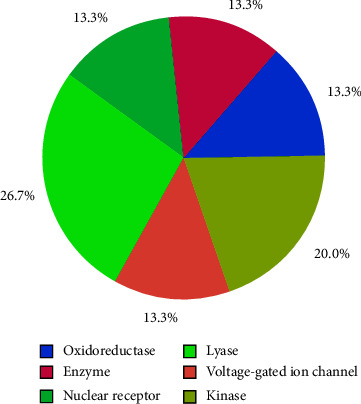
Pie charts of top targets predicted for wedelolactone by the Swiss target prediction server.

To predict human off-targets, estimate the possibility of crossreactions and evaluate the potential adverse effects of wedelolactone in humans, and molecular target prediction was performed using the Swiss Target Prediction server [30]. Molecular target prediction analysis revealed that lyase and kinase were the major protein targets for wedelolactone in humans (Figure 11). The prediction of kinase as one of the major targets is rather advantageous because several studies have revealed that human cellular kinases, such as Abelson tyrosine kinase (Abl), cyclin-dependent kinases (CDK), and numb-associated kinase (NAK), are directly involved in mediating the entrance, assembly, replication, and release of the SARS-CoV-2 virus [47, 48]. Thus, inhibition of these host cellular kinases is also significant for the inactivation of the virus.

### 3.4. Molecular Dynamics Simulation Studies

**Figure 12:**
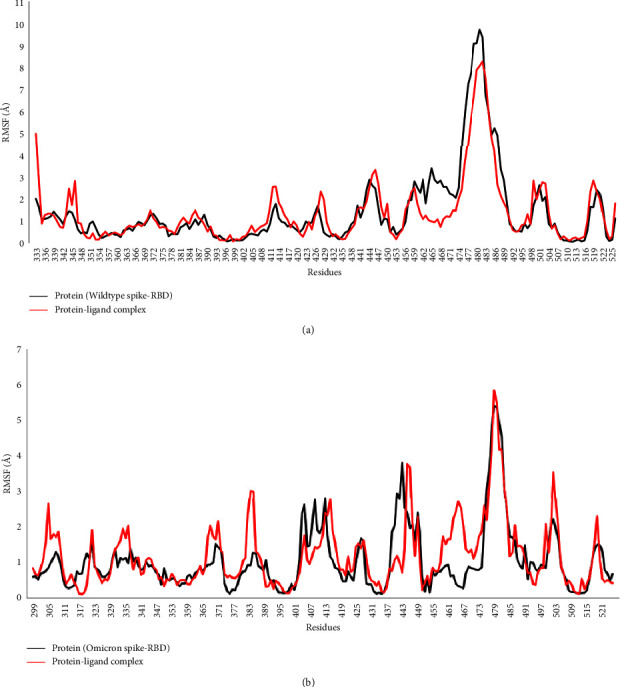
RMSF plots for ligand-free spike protein (black) and the spike-wedelolactone complex (red) of the SARS-CoV-2 (a) wild-type variant and (b) Omicron variant.

To further validate the results of molecular docking, the stability of the spike-wedelolactone complexes of wild-type and Omicron variants was analyzed and compared with the respective ligand-free spike proteins using MD simulation studies. The results of MD simulations were analyzed based on the RMSF values. RMSF measures the fluctuation and flexibility of individual residues in a protein during a simulation. Larger RMSF values suggest greater simulation flexibility, whereas lower RMSF values signify minimal conformational change, a firmly bound protein-ligand complex, and superior system stability [49]. The mean RMSF values for ligand-free spike proteins of wild-type and Omicron variants were 1.48 Å and 1.04 Å, respectively (Figure 12). In both of these ligand-free proteins, critical binding site residues (455, 493, 494, 496, 498, 501, and 505) had RMSF values below 3 Å. Likewise, the mean RMSF values for spike-wedelolactone complexes of wild-type and Omicron variants were 1.40 Å and 0.96 Å, respectively. Notably, the mean RMSF values for both of the ligand-free proteins were reduced upon binding with wedelolactone, indicating stable protein-ligand complexes. Moreover, the RMSF values for each critical binding site residue in S-RBDs of both of the ligand-free proteins were also found to be reduced (RMSF below 2.5 Å) in the respective protein-ligand complexes, suggesting strong binding interactions between these residues and wedelolactone [49]. Thus, MD simulation analysis revealed that wedelolactone was firmly docked in the binding sites of the respective spike proteins, possessing minimal conformational fluctuations and superior stability.

## 4. Conclusions

The spike proteins of SARS-CoV-2 and related coronaviruses have been established as a promising target for inhibiting viral entry into the host cells. The frequent emergence of new variants with an increasing number of mutations, especially in the spike-RBD regions, has limited the efficacies of vaccines and therapeutics currently available. Herein, the inhibition potential of several naturally occurring antiviral coumarins against the spike proteins of SARS-CoV-2 variants was investigated using computational methods. Among the 42 coumarins investigated, 26 displayed better binding energies ranging from −6.6 to −7.6 kcal/mol against the spike protein of the Omicron variant compared to two reference ligands: molnupiravir and ceftazidime. The top 10 best-docked coumarins, subjected to molecular docking studies against the spike proteins of other variants (wild-type, Alpha, Beta, Gamma, and Delta), also demonstrated decent binding energies (the best being −7.8 kcal/mol) through H-bonding and hydrophobic interactions. Physicochemical, QSAR, and pharmacokinetic analyses revealed wedelolactone as the best coumarin with optimal drug-likeness and minimal toxicity. MD simulation studies of spike-wedelolactone complexes validated stable binding of wedelolactone in the binding pocket of S-RBDs of wild-type and Omicron variants with reduced fluctuations (RMSF < 2.5 Å). In light of these findings, wedelolactone can be proposed for additional *in vitro* and *in vivo* clinical trials to further warrant its inhibition capabilities against the SARS-CoV-2 spike proteins [50, 51].

## Figures and Tables

**Table 1:** Binding interaction analysis of the top 10 best-docked coumarins and reference ligands with SARS-CoV-2 Omicron S-RBD.

S.N.	Compounds	Binding energy (kcal/mol)	Interacting residues	Interaction types	Interacting distance (Å)
1	Inophyllum C	−7.6	SER 494	Conventional H bond	1.95
			PHE 490	Pi-Pi stacked	4.87
			ARG 493	Pi-alkyl	4.38

2	(+)-Rutamarin	−7.5	TRP 436	Pi-Pi T-shaped	5.46, 5.74
			LYS 440	Alkyl	4.69
			PRO 373	Pi-alkyl	5.27, 5.33

3	Cordatolide A	−7.5	SER 494	Conventional H bond	2.07
			ARG 493	Pi-alkyl	3.77, 4.12

4	Soulattrolide	−7.5	SER 494	Conventional H bond	2.26
			PHE 490	Pi-Pi stacked	3.90, 5.27
			ARG 493	Pi-alkyl	5.21, 5.34

5	Wedelolactone	−7.4	TYR 449	Conventional H bond	2.31
			SER 494	Conventional H bond	2.35
			ARG 493	Pi-sigma, Pi-alkyl	3.73, (5.35)
			LEU 452	Pi-sigma	3.80, 5.14
			PHE 490	Pi-Pi stacked	5.33

6	Inophyllum A	−7.4	ASP 339	Pi-anion	4.03, 4.25
			LEU 371	Pi-alkyl	5.36
			LEU 368	Pi-alkyl	5.47

7	Inophyllum E	−7.4	SER 494	Conventional H bond	1.89
			PHE 490	Pi-Pi stacked	4.41, 5.84
			ARG 493	Pi-alkyl	4.75

8	(+)-Calanolide C	−7.3	SER 494	Conventional H bond	2.03, 2.51
			PHE 490	Pi-sigma	3.96
			ARG 493	Pi-alkyl	3.78, 4.13

9	Inophyllum B	−7.3	SER 494	Conventional H bond	2.43
			PHE 490	Pi-Pi stacked	3.85, 5.18
			ARG 493	Pi-alkyl	5.25, 5.28

10	Inophyllum D	−7.3	SER 494	Conventional H bond	1.88
			PHE 490	Pi-Pi stacked	4.27, 5.79
			ARG 493	Pi-alkyl	4.72

Reference ligands	Ceftazidime	−6.5	TYR 453	Conventional H bond	1.90
			ARG 403	Conventional H bond	2.45, 6.00
			SER 496	Conventional H bond	2.00
			HIS 505	Pi-cation, (C-H bond)	3.46, (4.08)
	Molnupiravir	−6.1	TYR 453	Conventional H bond	2.05
			ARG 403	Conventional H bond	2.20
			SER 496	C-H bond	3.33, 3.45
			ARG 493	C-H bond	3.46
			HIS 505	Pi-sigma	3.87

**Table 2:** Binding interaction analysis of the best-docked coumarins with spike proteins of SARS-CoV-2 variants among the selected top 10 coumarins.

SARS-CoV-2 variant S-RBDs	Best-dockedcoumarins	Binding energy (kcal/mol)	Interacting residues	Interaction types	Interacting distance (Å)
Wild-type variant PDB ID: 6M0J	Inophyllum B	−7.4	GLN 498	Conventional H bond	2.23
			ASN 501	Conventional H bond	2.86
			ARG 403	Pi-cation	4.91
			TYR 505	Pi-Pi T-shaped	4.91

Alpha variant PDB ID: 7EKF	Inophyllum D	−7.5	SER 494	Conventional H bond	2.26
			TYR 449	Pi-alkyl	5.17
			TYR 501	Pi-Pi stacked (Pi-Pi T-shaped)	5.60, (4.94)
			TYR 505	Pi-Pi stacked	3.77

Beta variant PDB ID: 7EKG	Soulattrolide	−7.2	GLN 493	Conventional H bond	2.18
			SER 494	Conventional H bond	2.82
			PHE 490	Pi-Pi stacked (Pi-alkyl)	4.60, 4.90, (4.77)
			LYS 484	Alkyl	4.81

Gamma variant PDB ID: 7EKC	Inophyllum E	−7.8	SER 494	Conventional H bond	2.32
			GLN 493	Carbon H bond	3.51
			LYS 484	Pi-Pi stacked	4.18, 5.52
			PHE 490	Pi-alkyl	4.99

Delta variant PDB ID: 7WBQ	Soulattrolide	−7.8	GLN 498	Conventional H bond	2.73
			ASN 501	Conventional H bond	2.94
			ARG 403	Pi-cation	4.03
			TYR 505	Pi-Pi T-shaped	5.72
			TYR 449	Carbon H bond	3.07

**Table 3:** Evaluation of Lipinski's RO5 and drug score for the top 10 best-docked coumarins and reference ligands.

S. No.	Compounds	Molecular weight (≤500)	Hydrogen bond donor (≤5)	Hydrogen bond acceptor (≤10)	CLogP (≤5)	Lipinski drug-likeness	Drug score
1	Inophyllum C	402.44	0	5	4.34	Yes	0.09
2	(+)-Rutamarin	356.41	0	5	4.02	Yes	0.15
3	Cordatolide A	342.39	1	5	3.17	Yes	0.14
4	Soulattrolide	404.46	1	5	4.11	Yes	0.10
5	Wedelolactone	314.25	3	7	2.09	Yes	0.30
6	Inophyllum A	404.46	1	5	4.11	Yes	0.10
7	Inophyllum E	402.44	0	5	4.34	Yes	0.09
8	(+)-Calanolide C	370.44	1	5	3.79	Yes	0.12
9	Inophyllum B	404.46	1	5	4.09	Yes	0.11
10	Inophyllum D	404.46	1	5	4.13	Yes	0.10
11	Molnupiravir	329.31	4	8	−1.16	Yes	0.18
12	Ceftazidime	546.58	3	11	−1.39	No	0.66

*Note.* Parenthetical figures indicate the threshold levels for Lipinski's RO5.

**Table 4:** ADMET analysis of the selected coumarins and reference ligands.

S. No.	Compounds	Absorption		Distribution	Metabolism	Excretion	Toxicity		
		HIA	P-gp I substrate/inhibitor	BBB	CYP3A4 substrate/inhibitor	Total clearance	Ames toxicity	Hepato toxicity	LD_50_ (oral rat acute toxicity)
1	Inophyllum C	100	No/yes	−0.45	Yes/yes	0.59	No	Yes	3.08
2	(+)-Rutamarin	97.86	No/yes	−0.27	Yes/yes	0.91	No	No	2.58
3	Cordatolide A	97.19	No/no	−0.13	Yes/no	0.57	Yes	No	2.92
4	Soulattrolide	97.64	Yes/yes	0.04	Yes/no	0.53	No	Yes	3.18
5	Wedelolactone	93.75	Yes/no	−1.35	Yes/no	0.56	No	No	2.41
6	Inophyllum A	97.64	Yes/yes	0.04	Yes/no	0.53	No	Yes	3.18
7	Inophyllum E	100	No/yes	−0.45	Yes/yes	0.59	No	Yes	3.08
8	(+)-Calanolide C	94.94	Yes/yes	−0.32	Yes/yes	0.51	No	No	2.53
9	Inophyllum B	97.64	Yes/yes	0.04	Yes/no	0.53	No	Yes	3.18
10	Inophyllum D	97.64	Yes/yes	0.04	Yes/no	0.53	No	Yes	3.18
11	Molnupiravir	53.46	No/no	−1.06	No/yes	0.20	No	Yes	2.16
12	Ceftazidime	16.74	Yes/no	−1.32	No/no	0.23	No	Yes	2.13

*Note.* HIA is expressed as %, BBB as a ratio (logBB), total clearance (log ml/min/kg), and LD_50_ (mol/kg).

## Data Availability

The data used to support the findings of this study are available from the corresponding author upon request.

## Abbreviations

SARS-CoV-2: Severe acute respiratory syndrome coronavirus 2
COVID-19: Coronavirus disease 2019
RBD: Receptor-binding domain
ACE-2: Angiotensin-converting enzyme 2
QSAR: Quantitative structure-activity relationship
ADMET: Absorption, distribution, metabolism, excretion, and toxicity
MD: Molecular dynamics
VOC: Variants of concern
PDB: Protein data bank
Å: Angstrom
RMSD: Root-mean-square deviation
RMSF: Root-mean-square fluctuation
SMILES: Simplified molecular input line entry system
RO5: Rule of five
ns: Nanosecond
2D: Two-dimensional
3D: Three-dimensional
HIA: Human intestinal absorption
BBB: Blood-brain barrier
P-gp I: Permeability-glycoprotein I
TPSA: Topological polar surface area.

## Supplementary Materials

Supplementary Materials Table S1: mutated amino acid residues in the spike-RBD of SARS-CoV-2 variants of concern in comparison to the wild-type variant. Table S2: selected natural coumarins with their reported antiviral activities. Table S3: binding energies of natural coumarins when docked against SARS-CoV-2 Omicron S-RBD. Figure S1: ChemDraw structures of selected natural coumarins.
