# Supplementary material for: In Silico Study of Coumarins: Wedelolactone as a Potential Inhibitor of the Spike Protein of the SARS-CoV-2 Variants
Source: J Trop Med. 2023 Feb 6;2023:4771745. doi: 10.1155/2023/4771745 (PMC11390206; doi:10.1155/2023/4771745)
Supplement: Supplementary Materials — Table S1: mutated amino acid residues in the spike-RBD of SARS-CoV-2 variants of concern in comparison to the wild-type variant. Table S2: selected natural coumarins with their reported antiviral activities. Table S3: binding energies of natural coumarins when docked against SARS-CoV-2 Omicron S-RBD. Figure S1: ChemDraw structures of selected natural coumarins. [file 4771745.f1.docx]

***In Silico* Study of Coumarins: Wedelolactone as a Potential Inhibitor of the Spike Protein of the SARS-CoV-2 Variants**

Saurav Katuwal^1^, Siddha Raj Upadhyaya^1^, Rishab Marahatha^1^, Asmita Shrestha^1^, Bishnu P. Regmi^2^, Karan Khadayat^1^, Saroj Basnet^3^, Ram Chandra Basnyat^1*^, and Niranjan Parajuli^1*^

*^1^Central Department of Chemistry, Tribhuvan University, Kirtipur, Kathmandu, Nepal*

*^2^Department of Chemistry, Florida Agricultural and Mechanical University, Tallahassee, Florida 32307, USA*

*^3^Center for Drug Design and Molecular Simulation Division, Cancer Care and Research Center, Kathmandu, Nepal*

^*^Corresponding authors: ram.basnyat@cdc.tu.edu.np (RCB); niranjan.parajuli@cdc.tu.edu.np (NP)

**Table S1.** Mutated amino acid residues in the spike-RBD of SARS-CoV-2 variants of concern in comparison to the wild-type variant <https://doi.org/10.3390/pathogens11010045>.

| **wild-type**  **variant** | **Variants of concern (VOCs)** | | | | |
| --- | --- | --- | --- | --- | --- |
|  | **Alpha**  **variant** | **Beta**  **variant** | **Gamma**  **variant** | **Delta**  **variant** | **Omicron variant** |
| G339 |  |  |  |  | G339D |
| S371 |  |  |  |  | S371L |
| S373 |  |  |  |  | S373P |
| S375 |  |  |  |  | S375F |
| K417 |  | K417N | K417T |  | K417N |
| N440 |  |  |  |  | N440K |
| G446 |  |  |  |  | G446S |
| L452 |  |  |  | L452R |  |
| S477 |  |  |  |  | S477N |
| T478 |  |  |  | T478K | T478K |
| E484 |  | E484K | E484K |  | E484A |
| Q493 |  |  |  |  | Q493R |
| G496 |  |  |  |  | G496S |
| Q498 |  |  |  |  | Q498R |
| N501 | N501Y | N501Y | N501Y |  | N501Y |
| Y505 |  |  |  |  | Y505H |

**Table S2.** Selected natural coumarins with their reported antiviral activities.

| **S.N.** | **Coumarins** | **Activity type** | **Activity value** | **Target site/Virus** | **Previously docked proteins** | **Citations** |
| --- | --- | --- | --- | --- | --- | --- |
| 1 | Inophyllum A | IC_50_ | 30 µM | HIV-1 reverse transcriptase | SARS coronavirus main protease | <https://doi.org/10.1016/j.sjbs.2020.11.038>  https://doi.org/10.1021/jm00078a001 |
| 2 | Inophyllum B | IC_50_ | 0.038 µM | HIV-1 reverse transcriptase | - | <https://doi.org/10.1021/jm00078a001> |
| 3 | Inophyllum C | IC_50_ | 10 µM | HIV-1 reverse transcriptase | - | <https://doi.org/10.1021/jm00078a001> |
| 4 | Inophyllum D | IC_50_ | 11 µM | HIV-1 reverse transcriptase | - | <https://doi.org/10.1021/jm00078a001> |
| 5 | Inophyllum E | IC_50_ | 100 µM | HIV-1 reverse transcriptase | - | <https://doi.org/10.1021/jm00078a001> |
| 6 | Inophyllum P | IC_50_ | 0.130 µM | HIV-1 reverse transcriptase | 3CLpro SARS-CoV-2 | <https://doi.org/10.1021/jm00078a001>  https://doi.org/10.1007/s11030-021-10230-6 |
| 7 | Eleutheroside B1 | IC_50_ | 64-125 µg/ml | Influenza A virus | - | https://doi.org/10.1016/j.biopha.2016.12.117 |
| 8 | Collinin | IC_50_ | 17.1 µg/ml | Hepatitis B virus (HBV) | SARS coronavirus main protease | <https://doi.org/10.1016/j.sjbs.2020.11.038>  https://doi.org/10.1016/S0031-9422(97)89023-1 |
| 9 | Wedelolactone | IC_50_ | 36.1 μM | Hepatitis C virus (HCV) | HCV NS5B polymerase  3CLpro of MERS-CoV & SARS-CoV | <https://doi.org/10.1007/s11030-021-10230-6>  https://doi.org/10.1093/nar/gkm1178 |
| 10 | Glycyrol | IC_50_ | 4.6 μg/mL | Hepatitis C virus (HCV) | - | https://doi.org/10.1111/1348-0421.12127 |
| 11 | Glycycoumarin | IC_50_ | 8.8 μg/mL | Hepatitis C virus (HCV) | 3CLpro of MERS-CoV, SARS-CoV & SARS-CoV-2 | <https://doi.org/10.1007/s11030-021-10230-6>  https://doi.org/10.1111/1348-0421.12127 |
| 12 | Glycyrin | IC_50_ | 7.2 μg/mL | Hepatitis C virus (HCV) | - | https://doi.org/10.1111/1348-0421.12127 |
| 13 | Heraclenol | EC_50_ | 0.115 μg/mL | HIV | - | https://doi.org/10.1016/S0031-9422(99)00554-3 |
| 14 | Heraclenin | IC_50_ | 20.1 μg/mL | HIV | - | https://doi.org/10.1016/S0031-9422(99)00554-3 |
| 15 | Osthol | IC_50_ | 11.7 μg/mL | HIV | - | https://doi.org/10.1016/S0031-9422(99)00554-3 |
| 16 | Psoralen | IC_50_  IC_50_ | 19.1 μg/mL  >200 μM | HIV  SARS-CoV 3CL^pro^ | - | <https://doi.org/10.3109/14756366.2014.1003215>  https://doi.org/10.1248/cpb.49.877 |
| 17 | Conferol | IC_50_ | 0.26-0.86 μg/mL | H1N1 virus | - | https://www.plantsjournal.com/archives/?year=2020&vol=8&issue=2&part=B&ArticleId=1120 |
| 18 | Kellerin | EC_50_ | 38 μg/mL | Herpes  simplex virus 1 (HSV-1) | - | https://pubmed.ncbi.nlm.nih.gov/25237347/ |
| 19 | Isomesuol | IC_50_ | 2-2.5 μM | HIV | SARS coronavirus main protease  3CLpro of MERS-CoV | <https://doi.org/10.1016/j.sjbs.2020.11.038>  <https://doi.org/10.1007/s11030-021-10230-6>  https://doi.org/10.1016/j.antiviral.2005.02.006 |
| 20 | Disparinol A | IC_50_ | 0.5 μM | HIV | - | https://doi.org/10.1016/j.bmcl.2005.07.041 |
| 21 | Esculetin | ED_50_ | 2.51 μg/mL | HIV | - | https://doi.org/10.1016/S0968-0896(00)00225-X |
| 22 | (+)-Calanolide A | IC_50_ | 20 μM | HIV | SARS coronavirus main protease | <https://doi.org/10.1016/j.sjbs.2020.11.038>  https://doi.org/10.1021/jm00093a004 |
| 23 | (+)-Calanolide B | IC_50_ | 15 μM | HIV | - | https://doi.org/10.1021/jm00093a004 |
| 24 | (+)-Calanolide C | IC_50_ | 30 μM | HIV | - | https://doi.org/10.1021/jm00093a004 |
| 25 | Bergapten | IC_50_  IC_50_ | 24.8 μg/mL  >200 μM | HIV  SARS-CoV 3CL^pro^ | - | https://doi.org/10.3109/14756366.2014.1003215  https://doi.org/10.1248/cpb.49.877 |
| 26 | Saxalin | IC_50_ | 26.3 μg/mL | HIV | - | https://doi.org/10.1248/cpb.49.877 |
| 27 | Leptodactylone | EC_50_ | <100 μg/ml | SARS-CoV | - | https://doi.org/10.1080/10286020500382397 |
| 28 | Suksdorfin | EC_50_ | 2.6 ± 2.1 μM | HIV | SARS coronavirus main protease | <https://doi.org/10.1016/j.sjbs.2020.11.038>  https://doi.org/10.1016/S0968-0896(00)82054-4 |
| 29 | Toddacoumaquinone | EC_50_ | 10 μg/mL | Herpes  simplex virus | - | https://doi.org/10.1248/cpb.43.1039 |
| 30 | Cordatolide A | IC_50_ | 12.3 μM | HIV-1 reverse transcriptase | SARS coronavirus main protease | <https://doi.org/10.1016/j.sjbs.2020.11.038>  https://doi.org/10.1055/s-2006-957483 |
| 31 | Cordatolide B | IC_50_ | 19.0 µM | HIV-1 reverse transcriptase | - | https://doi.org/10.1055/s-2006-957483 |
| 32 | Soulattrolide | IC_50_ | 0.81µM | HIV-1 reverse transcriptase | - | https://doi.org/10.1055/s-2006-957483 |
| 33 | Oxypeucedanin | IC_50_ | 23.4 μg/mL | HIV | 3CLpro of SARS-CoV-2 | <https://doi.org/10.1007/s11030-021-10230-6>  https://doi.org/10.1016/S0031-9422(99)00554-3 |
| 34 | Imperatorin | EC_50_ | <0.10 μg/mL | HIV | - | https://doi.org/10.1016/S0031-9422(99)00554-3 |
| 35 | (+)-Rutamarin | EC_50_ | 1.62 μM | Herpes  simplex virus | ATPase domain of human topoisomerase IIα  SARS coronavirus main protease | <https://doi.org/10.1016/j.sjbs.2020.11.038>  https://doi.org/10.1128/AAC.01259-13 |
| 36 | Isoobtusitin | IC_50_ | 2.9 μM | Poliovirus | - | https://doi.org/10.1248/cpb.49.619 |
| 37 | Mesuol | IC_50_ | 2 - 2.5 μM | HIV | SARS coronavirus main protease  3CLpro of SARS-CoV-2 | <https://doi.org/10.1016/j.sjbs.2020.11.038>  https://doi.org/10.1007/s11030-021-10230-6  https://doi.org/10.1016/j.antiviral.2005.02.006 |
| 38 | Chalepin | IC_50_ | 1.7 ± 0.5 μg/mL | Hepatitis C virus (HCV) | - | https://doi.org/10.1016/j.fitote.2014.10.011 |
| 39 | Isomammeigin | EC_50_ | 9.6 μg/mL  10.7 μg/mL | Dengue virus  Chikungunya virus | - | https://doi.org/10.1186/s12906-017-1562-1 |
| 40 | Mammein | EC_50_ | 2.6 μg/mL  0.5 μg/mL | Dengue virus  Chikungunya virus | - | https://doi.org/10.1186/s12906-017-1562-1 |
| 41 | Xanthotoxin | IC_50_ | >200 μM    >200 μM | SARS-CoV 3CL^pro^  SARS-CoV PL^pro^ | - | https://doi.org/10.3109/14756366.2014.1003215 |
| 42 | Isopimpinellin | IC_50_ | >200 μM    <200 μM | SARS-CoV 3CL^pro^  SARS-CoV PL^pro^ | - | https://doi.org/10.3109/14756366.2014.1003215 |

**Table S3.** Binding energies of natural coumarins when docked against SARS-CoV-2 Omicron S-RBD.

| **S.N.** | **Coumarins** | **Binding energy (kcal/mol)** | **S.N.** | **Coumarins** | **Binding energy (kcal/mol)** |
| --- | --- | --- | --- | --- | --- |
| 1 | Inophyllum C | **-7.6** | 23 | Toddacoumaquinone | **-6.7** |
| 2 | (+)-Rutamarin | **-7.5** | 24 | Mesuol | **-6.7** |
| 3 | Cordatolide A | **-7.5** | 25 | Saxalin | **-6.7** |
| 4 | Soulattrolide | **-7.5** | 26 | Oxypeucedanin | **-6.6** |
| 5 | Wedelolactone | **-7.4** | 27 | Isomesuol | -6.3 |
| 6 | Inophyllum A | **-7.4** | 28 | Disparinol A | -6.3 |
| 7 | Inophyllum E | **-7.4** | 29 | Heraclenin | -6.2 |
| 8 | (+)-Calanolide C | **-7.3** | 30 | Eleutheroside B1 | -6.0 |
| 9 | Inophyllum B | **-7.3** | 31 | Heraclenol | -6.0 |
| 10 | Inophyllum D | **-7.3** | 32 | Mammein | -6.0 |
| 11 | (+)-Calanolide B | **-7.2** | 33 | Imperatorin | -6.0 |
| 12 | Cordatolide B | **-7.2** | 34 | Psoralen | -6.0 |
| 13 | Inophyllum P | **-7.2** | 35 | Esculetin | -5.9 |
| 14 | Kellerin | **-7.1** | 36 | Isoobtusitin | -5.8 |
| 15 | (+)-Calanolide A | **-7.1** | 37 | Suksdorfin | -5.7 |
| 16 | Glycyrol | **-7.0** | 38 | Xanthotoxin | -5.7 |
| 17 | Conferol | **-6.9** | 39 | Leptodactylone | -5.7 |
| 18 | Chalepin | **-6.9** | 40 | Osthol | -5.7 |
| 19 | Isomammeigin | **-6.9** | 41 | Bergapten | -5.6 |
| 20 | Collinin | **-6.8** | 42 | Isopimpinellin | -5.5 |
| 21 | Glycycoumarin | **-6.7** | 43 | Molnupiravir* | -6.1 |
| 22 | Glycyrin | **-6.7** | 44 | Ceftazidime* | -6.5 |
|  |  |  | *Reference ligands. Binding energies lower than that of reference ligands are shown in bold. | | |


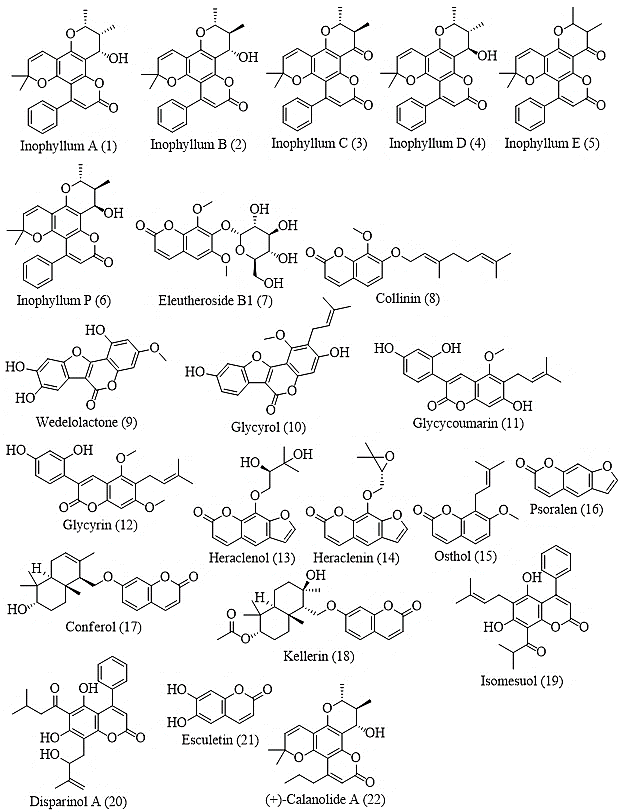


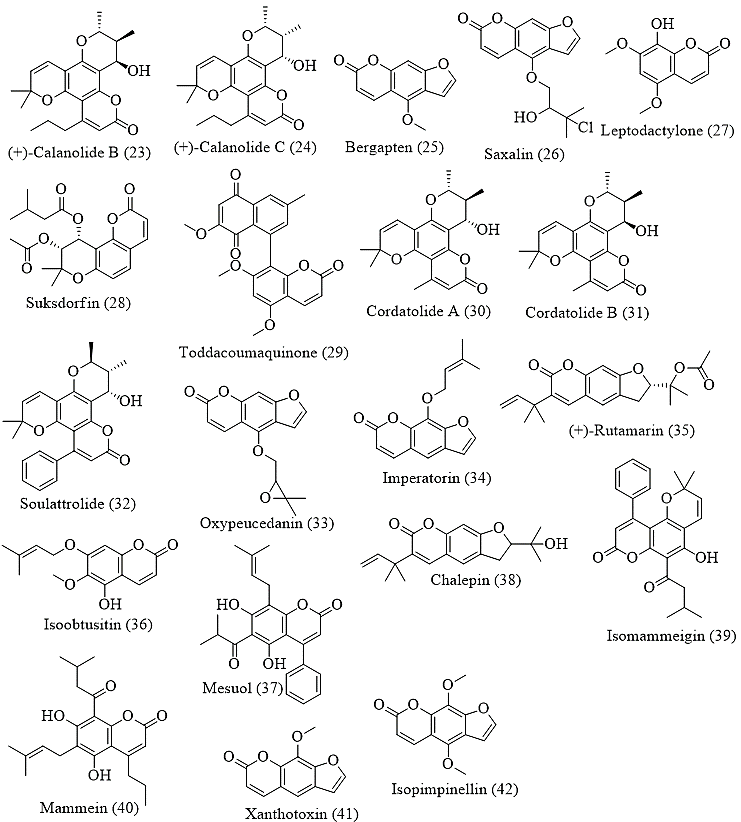


Figure S1. ChemDraw structures of selected natural coumarins.
